# Activating Striatal Parvalbumin Interneurons to Alleviate Chemotherapy‐Induced Muscle Atrophy

**DOI:** 10.1002/jcsm.13782

**Published:** 2025-04-08

**Authors:** Jun Hu, Jingyuan Liu, Yuqing Yan, Ziyu Shen, Junlong Sun, Yongjun Zheng

**Affiliations:** ^1^ Department of Pain, Huadong Hospital Fudan University Shanghai China; ^2^ Shanghai Key Laboratory of Clinical Geriatric Medicine Fudan University Shanghai China; ^3^ School of Exercise and Health Shanghai University of Sport Shanghai China; ^4^ Department of Rehabilitation Medicine Shanghai University of Medicine & Health Sciences Affiliated Zhoupu Hospital Shanghai China

**Keywords:** cisplatin, muscle atrophy, parvalbumin interneurons, striatum

## Abstract

**Background:**

Cisplatin is a widely used chemotherapeutic agent for treating solid tumours. Still, it induces severe side effects, including muscle atrophy. Understanding the mechanisms of cisplatin‐induced muscle loss and exploring potential therapeutic strategies are essential. Parvalbumin (PV) interneurons in the striatum play a crucial role in motor control, and recent studies suggest that their activation may alleviate motor deficits. This study investigates the effects of chemogenetic activation of PV interneurons on cisplatin‐induced muscle atrophy and motor dysfunction in mice.

**Methods:**

Wild‐type C57BL/6 mice and transgenic hM3Dq mice were used in this study. Cisplatin (3 mg/kg) was administered intraperitoneally for 7 days to induce muscle atrophy. Mice were then treated with clozapine‐n‐oxide (CNO) to activate PV interneurons. Muscle strength and endurance were assessed using grip strength measurements, the inverted grid test and the wire hang test. Neuromuscular junction (NMJ) integrity was examined via histological analysis. Exercise intervention was also included, using a treadmill with a 15° incline for 60 min at varying speeds during seven consecutively days.

**Results:**

Cisplatin treatment significantly reduced body weight (*p* < 0.001), grip strength (forelimb strength: *p* < 0.001, four‐limb strength: *p* < 0.001), endurance (inverted grid test: *p* = 0.047, wire hang test: *p* = 0.014) and NMJ integrity (partially innervated NMJs: *p* = 0.0383). PV interneuron activation with CNO improved spontaneous motor activity in cisplatin‐treated mice, as evidenced by a significant increase in total travel distance (*p* = 0.049) in the open‐field test. Histological analysis showed a reduced ratio of partially innervated NMJs in the PV‐cre group compared to the control virus group (*p* = 0.0441). Muscle strength also improved significantly, with forelimb grip strength increased (*p* < 0.001) and four‐limb grip strength increased (*p* = 0.018). Muscle wet‐weight ratios were significantly higher in the PV‐cre group (quadriceps: *p* = 0.030). Exercise intervention significantly improved grip strength (forelimb: *p* < 0.001, four‐limb: *p* = 0.002), muscle endurance (four‐limb hang test: *p* = 0.048) and muscle weight (quadriceps: *p* = 0.015, gastrocnemius: *p* = 0.022), with an increase in muscle fibre cross‐sectional area (*p* = 0.0018).

**Conclusion:**

Activation of PV interneurons significantly alleviates cisplatin‐induced motor deficits and muscle atrophy by improving spontaneous motor activity, NMJ integrity and muscle function. It has a similar effect to short‐term exercise and may offer a promising therapeutic strategy for mitigating chemotherapy‐induced muscle atrophy.

## 1 Introduction

Chemotherapy necessitates the standardized protocols of anticancer drugs tailored to each type of cancer. Cisplatin, a platinum‐based anticancer drug, is widely used in the treatment of solid tumours. While effective in combating tumours, cisplatin also causes toxic effects, including nausea, weight loss and muscle atrophy [1]. These symptoms are markers of cachexia and sarcopenia, significantly impacting clinical outcomes. Given the severe side effects of cisplatin, understanding its impacts on muscle loss is essential.

Research indicates that the rate of muscle loss in chemotherapy patients is significantly reduced [2]. Direct injection of cisplatin into healthy mice has been shown to increase levels of muscle atrophy factors, leading to weight loss, skeletal muscle atrophy and weakness [1]. Muscle atrophy is a negative predictor of treatment outcomes in cancer patients, often correlated with increased cancer‐related mortality. Moreover, muscle atrophy exacerbates tumour‐induced pain, weakness and cachexia, creating a vicious cycle that negatively affects the patient's quality of life [3]. Therefore, identifying methods to prevent and mitigate muscle loss and atrophy during cisplatin treatment is crucial.

Various interventions have been explored to counteract muscle atrophy. Current treatments for muscle atrophy include exercise, nutritional and pharmacological interventions [4, 5, 6]. Exercise training effectively enhances muscle strength, improves muscle function and promotes muscle hypertrophy [7]. Motor activity originates in the primary motor cortex (M1) and is transmitted via the corticospinal tract to the anterior horn of the spinal cord, subsequently controlling muscle movement. The coordination between the pyramidal and extrapyramidal systems constitutes overall motor function.

The striatum, a crucial component of the extrapyramidal system, plays a significant role in regulating motor homeostasis and muscle tone [8]. Functionally, the striatum acts as a critical relay linking the cerebral cortex to subcortical structures, transforming cortical input into output signals that guide and modulate behaviour [9]. Within the striatum, the predominant neuronal type is the medium spiny neuron (MSN), comprising approximately 95% of striatal neurons [10]. The remaining 5% consists of various interneuron types, among which the parvalbumin (PV)‐expressing interneurons are notably distinctive [11]. PV interneurons, characterized by their GABAergic properties, play an important regulatory role by modulating the activity of neighbouring neurons, thereby supporting fine motor control and ensuring smooth and coordinated movement [12, 13]. Recent advancements using single‐cell calcium imaging combined with optogenetic techniques reveal that the striatum receives long‐range projections from cortical PV interneurons, and optogenetic manipulation of cortical interneurons showed that their state critically influences motor activity [14]. Another study directly intervened with striatal interneurons, revealing that optogenetic activation of PV interneurons in the striatum can trigger a transition from rest to movement in mice [15]. However, the long‐term effects of neuronal activation and its potential to improve muscle atrophy are unknown.

In this study, we examined the effects of chemogenetic activation of PV neurons and further investigated its anti‐atrophic effects on cisplatin‐induced muscle loss.

## 2 Methods

### 2.1 Experimental Animals

Wild‐type male C57BL/6 mice for 8 weeks were obtained from Shanghai Biomodel Organism Co. Ltd. Transgenic hM3Dq (C57BL/6Smoc‐Gt (ROSA)26Sor^em1(CAG‐LSL‐HA‐GqDREADD(hM3Dq)‐P2A‐mCitrine‐WPRE‐polyA)Smoc^) mice were acquired from The Jackson Laboratory (JAX: 026942). All mice were housed under controlled environmental conditions (21°C–25°C, 40%–50% humidity, 12‐h light/dark cycle) with free access to food and water at the Animal Facility of Fudan University. To ensure randomization, all animals were randomly assigned to experimental groups. Based on previous studies, cisplatin was administered intraperitoneally at a dose of 3 mg/kg body weight daily for 7 days [1]. Body weight was measured daily, and after the seven‐day cisplatin administration period, behavioural tests were conducted to assess muscle atrophy. The study was approved by the Ethical Review Board of the Department of Animal Science, Fudan University and was conducted in accordance with the appropriate ethical guidelines (2024‐HDYY‐062).

### 2.2 Drug Administration

Cisplatin (232 120; Sigma‐Aldrich) was freshly dissolved in phosphate‐buffered saline before administration to the mice. The control group received the same solvent without cisplatin. For chemogenetic studies, all mice were injected with clozapine‐n‐oxide [16] (CNO) (1.0 mg/kg, i.p. Abcam, ab141704), at two different time (9:00, 21:00) for seven consecutively days.

### 2.3 Behavioural Tests

Various behavioural tests were conducted to evaluate muscle strength and endurance.

### 2.4 Grip Strength Measurement

Grip strength was evaluated using a grip strength meter (KW‐ZL; KEW BASIS, China). Each trial involved placing the mouse on the device and pulling it parallel to the ground until the mouse released its grip. Five trials were conducted per time point, with a 10‐min rest between trials to ensure muscle recovery. The average of the five trials was calculated to obtain the final grip strength value for each mouse.

### 2.5 Inverted Grid Test

Grip endurance was assessed using an inverted grid device with a 10 × 30 cm metal wire grid placed 20 cm above an open box. The grid was rotated upside down allowing mice to move freely. Mice were timed until they released their grip and fell or reached the maximum grip duration of 240 s. Each time point involved two trials, with the best trial scored. If a mouse fell from the mesh grid within 15 s, additional attempts were allowed (max: three attempts each trial) within an interval of 1 min.

### 2.6 Wire Hang Test

A standard linear wire hang apparatus, consisting of a 30 cm long, 20 cm high metal wire, was used to assess grip endurance. Mice were placed on the wire with their limbs to support themselves against falling and moving along the wire. Mice were timed until they released their grip and fell or reached the maximum hang duration of 180 s. Each time point involved two trials, with the best trial scored. If a mouse fell from the wire within 15 s, additional attempts were allowed (max: three attempts each trial) within an interval of 1 min.

### 2.7 Open‐Field Test

An open‐field test was used to evaluate motor ability and anxiety. Mice were acclimated to the testing environment for 1 h before being placed in the same location within a polyvinyl chloride box (40 × 40 × 50 cm). Mouse activity was recorded by a video behaviour analysis system (EthoVisionXT; Noldus Information Technology, Netherlands). Motor function was determined by calculating the total distance travelled. The PVC box was cleaned with 75% alcohol between tests to remove odours.

### 2.8 Stereotaxic Injection

For targeted neuronal activation, rAAV‐PV‐CRE‐bGH polyA (PT‐0275; Shumi Brain Science and Technology Co. Ltd. China) and control virus were stereotaxically injected into the striatum. Mice were anaesthetised with isoflurane (1%–2.5%) and fixed in a stereotaxic apparatus. The head was disinfected, the skin incised and the skull surface cleaned with hydrogen peroxide. Bregma was used as the origin point for levelling the mouse brain. A total of 600 nL virus was injected into the bilateral striatum (AP = 0.6 mm, ML = ± 1.7 mm, DV = − 3 mm). Additionally, the virus efficiency in targeting PV interneurons was validated. We microinjected into the striatum with 500 nL viral cocktails (1:1) of rAAV‐PV‐CRE‐bGH polyA and rAAV2/9‐Ef1a‐DIO‐hM3D(Gq)‐mCherry‐WPRE (PT‐0042; Shumi Brain Science and Technology Co. Ltd. China). PV neurons in the striatum were marked with immunostaining, and calculating the colocalization of mCherry with PV protein (Figure S4).

### 2.9 Exercise Intervention

To further assess the effects on muscle function, a treadmill (DSPT‐216) set at a 15° incline was used. The belt speed started at 6 m/min for 60 s, then increased to 16 m/min over 5 min and maintained that speed until the end of the 60‐min intervention. This exercise was conducted at two different time (9:00, 21:00).

### 2.10 Haematoxylin–Eosin (HE) Staining

After behavioural tests, mice were euthanized with CO_2_ and perfused with 4% paraformaldehyde (PFA). Muscle tissues were harvested, paraffin‐embedded, sectioned at 5 μm and stained with HE to examine pathological changes in muscle fibres.

### 2.11 Immunohistochemistry

Mice were anaesthetised with isoflurane and perfused with PBS, followed by 20 mL 4% PFA. The brains were dissected and post‐fixed in 4% PFA at 4°C overnight and dehydrated using 30% sucrose buffer and then sectioned at 40 μm using a freezing microtome (RWD, FS800A). TA muscles were teased into fibres for whole‐mount staining. Tissues of control and experimental groups were pasted on the same glass slide to maintain uniform conditions during the staining and image collection processes. For immunostaining, sections or muscle fibres were treated with the immunostaining buffer (1% BSA, 0.5% Triton X‐100 in PBS) for 1 h at room temperature and then incubated with primary antibodies at 4°C overnight. After washing three times with PBS, sections or muscle fibres were incubated with fluorescence‐conjugated secondary antibodies (Jackson Immunology) at 37°C for 45 min. Fluorescence images were captured by the Leica DMi8 Confocal microscope and analysed using ImageJ software.

The primary antibodies used in immunostaining were as follows: anti‐parvalbumin Antibody (Swant, Cat# PV25, RRID: AB_10000344), anti‐Neurofilament (Cell Signalling Technology, Cat# 2837S, RRID: AB_823575) and anti‐Synapsin‐1 (Cell Signalling Technology, Cat# 5297S, RRID: AB_AB_2616578).

### 2.12 Statistical Analysis

Data analysis and graphing were performed using GraphPad Prism 9.5 software. Normal distribution was assessed using the Shapiro–Wilk test, and comparisons between the two groups were performed using an unpaired *t*‐test. One‐way ANOVA analysis followed by post hoc Tukey's test was used for weight analysis. A significance threshold of *p* < 0.05 was set. Each data point represents one mouse, with all data presented as Mean ± SEM.

## 3 Results

### 3.1 Cisplatin Induces Severe Muscle Atrophy in Mice

**FIGURE 1:**
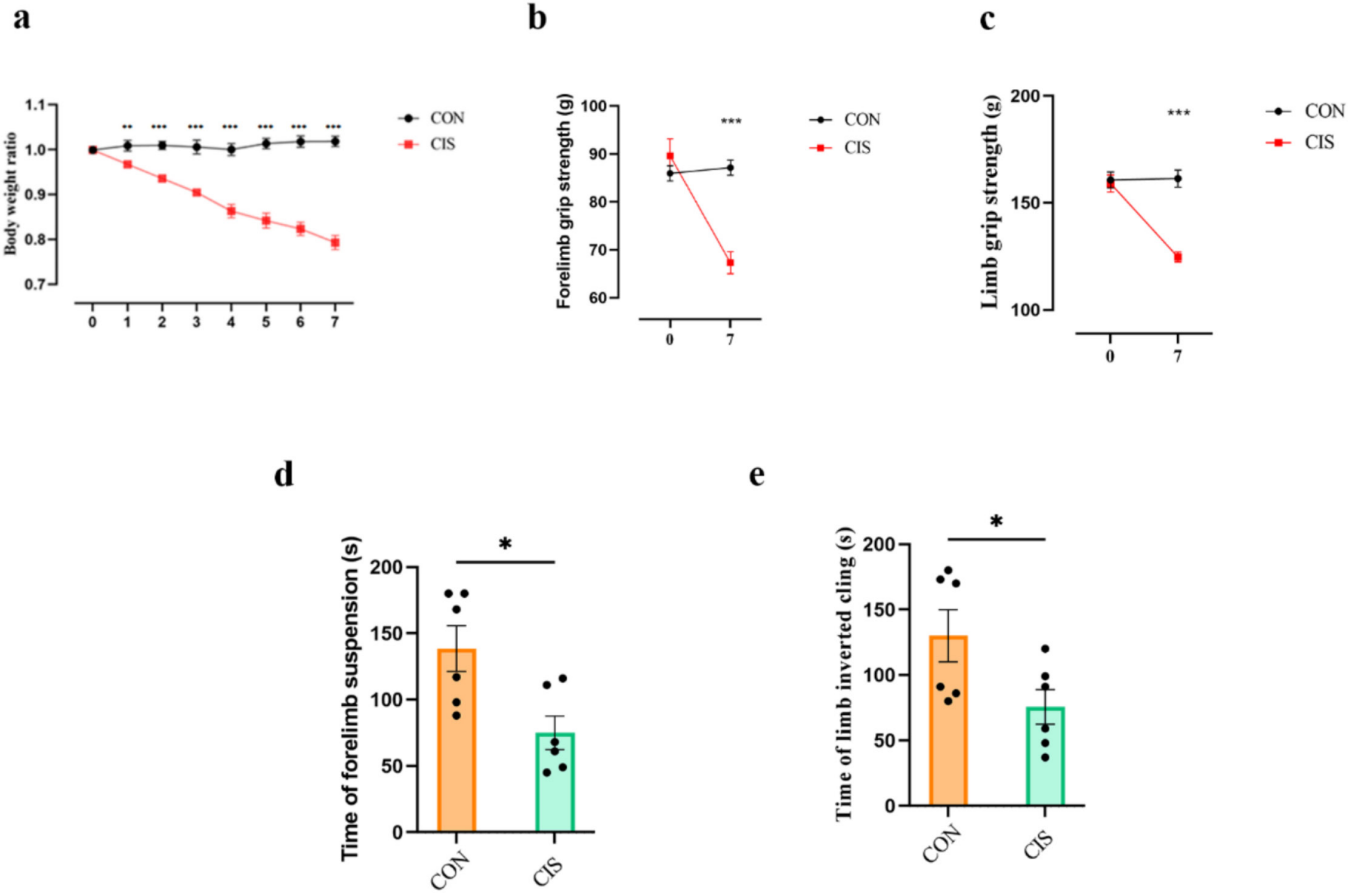
Effects of cisplatin on muscle atrophy. (a) Reduced body weight ratio (measured weight/initial weight) following cisplatin treatment. (b) Forelimb strength and (c) hindlimb strength were assessed using a grip strength meter, measured in grams before and after cisplatin injection. (d) The forelimb function and (e) hindlimb function were evaluated by measuring the forelimb hang time in seconds before and post‐injection. Data are presented as Mean ± SEM (*n* = 6). CON: control group; CIS: cisplatin‐treated group.

Cisplatin, a widely used chemotherapeutic agent, is known for its efficacy in treating solid tumours but often induces severe side effects, including muscle atrophy [17]. To investigate its impact, we established a cisplatin‐induced muscle atrophy model in mice. Behavioural and histological analyses confirmed that cisplatin treatment induces significant muscle atrophy in mice. On the seventh day post‐model establishment, cisplatin‐treated (CIS) mice exhibited a marked reduction in body weight compared to the control (CON) group (Figure 1a). Grip strength measurements showed significant reductions in both forelimb (Unpaired *t*‐test, *t*_10_ = 5.299, *p* < 0.001; Figure 1b) and four‐limb strength (Unpaired *t*‐test, *t*_10_ = 7.882, *p* < 0.001; Figure 1c) in the CIS group. Additionally, muscle function tests indicated shorter duration on the forelimb hang tests (Unpaired *t*‐test, *t*_10_ = 2.270, *p* = 0.047; Figure 1d) and inverted grid (Unpaired *t*‐test, *t*_10_ = 2.964, *p* = 0.014; Figure 1e) for the CIS group, demonstrating motor deficits and reduced endurance. These findings are further supported by the detailed histological data provided in Figure S1. In the open‐field test, CIS mice displayed significantly reduced spontaneous motor activity compared to the CON group. Specifically, the CIS group showed a marked decrease in total travel distance (Unpaired *t*‐test, *t*_10_ = 7.469, *p* < 0.001; Figure S2b), suggesting impaired motor function following cisplatin administration. Given cisplatin's neurotoxic effects, the mechanisms underlying observed muscle atrophy and motor deficits remain incompletely understood [17]. It is unclear whether these deficits stem from direct toxicity to muscle fibres or involve broader damage to the nervous system, such as the peripheral nervous system and neuromuscular junctions (NMJs), which play a key role in muscle force and somatomotor control [18]. We observed significant NMJ dysfunction in CIS mice, including a pronounced increase of partially innervated NMJs (Unpaired *t*‐test, *t*_*1*0_ = 2.385, *p* = 0.0383; Figure S3), with the rising trend of acetylcholine receptor (AChR) fragmentation and denervated NMJs. These structural disruptions establish a mechanistic link between cisplatin‐induced neurotoxicity and behavioural impairments, such as reduced muscle strength and autonomous movement.

### 3.2 Impact of PV Interneuron Activation on Motor Function and NMJ Integrity in Cisplatin‐Treated Mice

**FIGURE 2:**
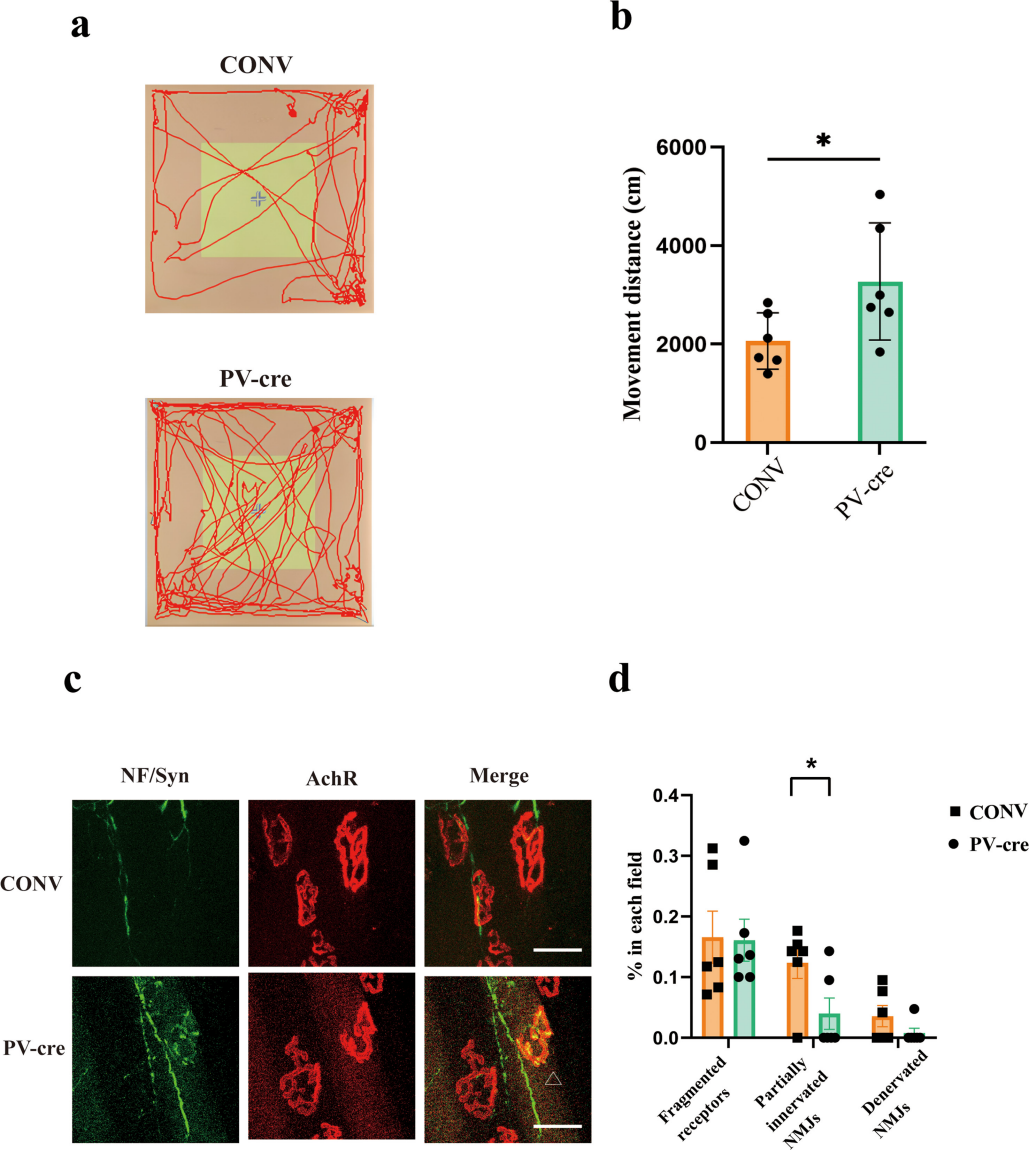
Impact of striatal PV neuron intervention on motor and emotional states in cisplatin‐treated mice. (a) Movement trajectories following activation of striatal PV interneurons. (b) Spontaneous motor activity is quantified by total travel distance (cm) within a fixed period. (c) Representative images of NMJs in the TA muscle. The muscle fibres were stained whole mount with α‐BTX (red) to label AChR clusters and NF/synapsin‐1 (green) to label nerve terminals. Scale bar, 50 μm. (d) Quantification of NMJs in (c), including the ratio of fragmented receptors, partially innervated NMJs and denervated NMJs in each 20 × field. CONV group, *n* = 105 NMJs from three mice; PV‐cre group, *n* = 135 NMJs from three mice. Data are presented as Mean ± SEM (*n* = 6). CONV: control virus injection; PV‐cre: PV‐cre virus injection.

**FIGURE 3:**
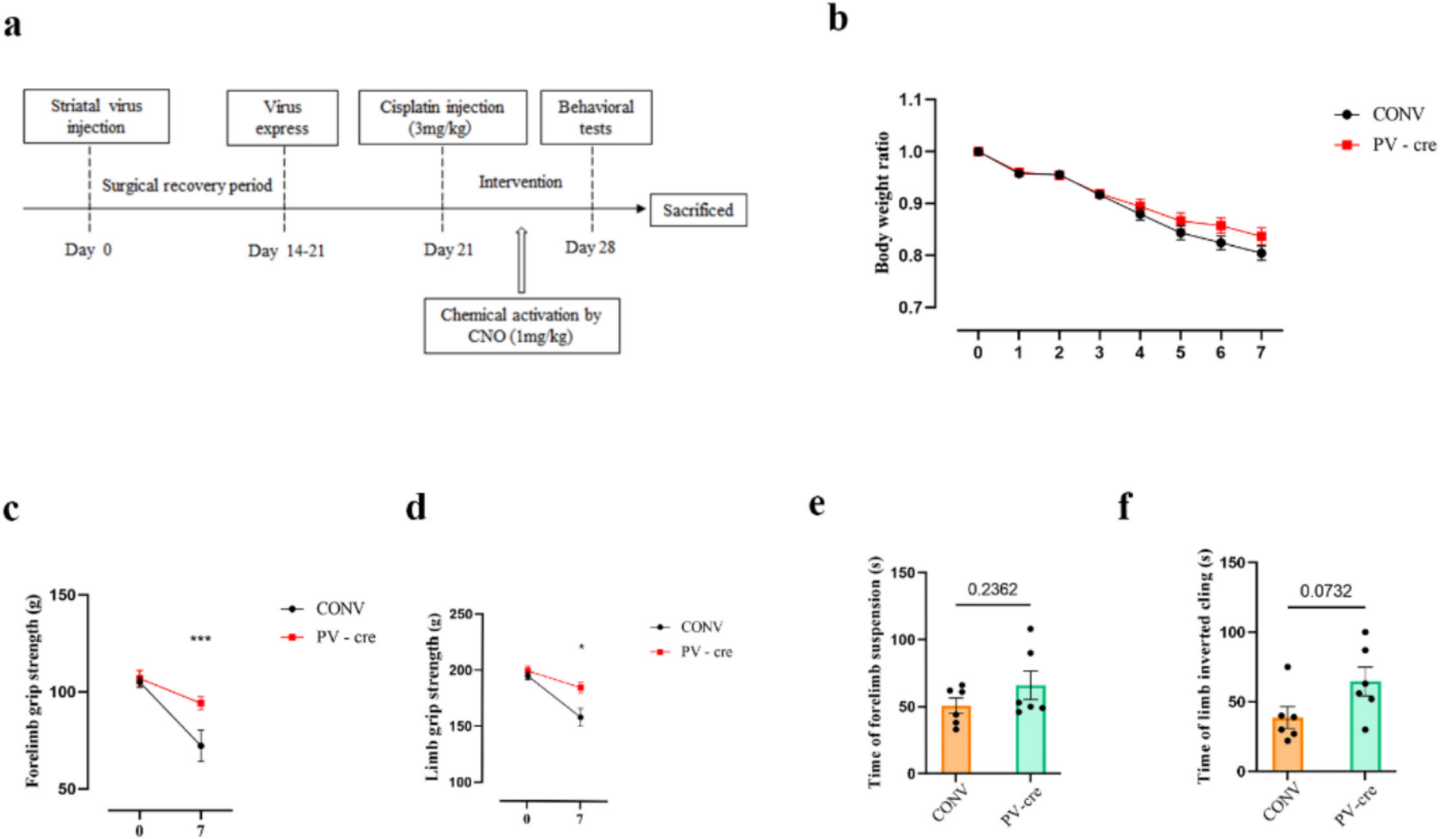
Chemogenetic activation of striatal PV interneurons on cisplatin‐induced muscle atrophy in vivo. (a) Schematic of the striatal interneuron intervention protocol. (b) Body weight ratio (measured weight/ initial weight) following chemogenetic activation in cisplatin‐treated mice. (c) Forelimb grip strength and (d) hindlimb grip strength were assessed before and 7 days after chemogentic activation. (e) Forelimb function and (f) hindlimb function evaluated by forelimb hang time. Data are presented as Mean ± SEM (*n* = 6). CONV: control virus injection; PV‐cre: PV‐cre virus injection.

PV interneurons in the striatum play a crucial role in regulating motor circuits and emotional behaviour [15]. To evaluate their potential therapeutic role, we employed chemogenetic activation using CNO 3 weeks post‐viral injection. In short, we injected the rAAV‐PV‐CRE‐bGH polyA virus into the bilateral striatum of hM3Dq mice. Following a three‐week incubation period, PV interneurons in the striatum specifically express Cre recombinase, which can combine with hM3Dq receptor in hM3Dq mice. The hM3Dq receptor triggers intracellular signalling pathways leading to neuronal activation when bound to its ligand, such as CNO. Mice underwent daily CNO administration following the establishment of cisplatin‐induced muscle atrophy (Figure 3a). Chemogenetic activation of PV interneurons significantly alleviated behavioural deficits in the open‐field test of mice in the rAAV‐PV‐CRE‐bGH polyA virus injection (PV‐cre) group. Compared to the control virus injection (CONV) group, PV‐cre mice exhibited increased total travel distance (Unpaired *t*‐test, *t*_10_ = 2.240, *p* = 0.049; Figure 2b). This finding indicates that the activation of PV interneuron enhances spontaneous motor activity in cisplatin‐treated mice. Additionally, the virus efficiency in targeting PV interneurons was validated in our model, ensuring the robustness of chemogenetic activation (Figure S4).

Previous studies have highlighted NMJ dysfunction as a key contributor to muscle atrophy and motor impairments, with evidence suggesting that interventions targeting NMJ integrity can mitigate these effects [19]. For instance, exercise has been shown to improve NMJ function, and exercise‐related therapies could potentially attenuate the progression of neuromuscular degeneration [20]. So we checked the effect of activating PV interneurons on NMJ dysfunction. After activating PV neurons, the ratio of partially innervated NMJs was significantly reduced, with the amelioration of denervation (Unpaired *t*‐test, *t*_10_ = 2.302, *p* = 0.0441; Figure 2c–d, indicated by the triangle is representative amelioration of partially innervated NMJ), suggesting protective effects of PV interneuron activation on NMJ integrity. These effects underscore the dual role of PV interneurons in restoring both central motor regulation and peripheral neuromuscular coordination.

In summary, chemogenetic activation of PV interneurons represents a promising therapeutic strategy to mitigate cisplatin‐induced neuromuscular impairments. These findings provide a foundation for future investigations into integrating central and peripheral interventions to combat chemotherapy‐induced motor deficits.

### 3.3 Chemogenetic Activation of PV Interneurons Improves Muscle Atrophy in Cisplatin‐Treated Mice

Building on the observed motor improvement, we next assessed the impact of PV interneuron activation on muscle atrophy. Given that the cisplatin‐treated (CIS) group exhibited severe muscle atrophy, as evidenced by reduced grip strength, decreased muscle wet weight and significant muscle fibre degradation, it was crucial to determine whether activating PV interneuron could mitigate these effects.

Behavioural assessments revealed that PV interneuron activation significantly improved forelimb grip strength (Unpaired *t*‐test, *t*_10_ = 6.136, *p* < 0.001; Figure 3c) and four‐limb grip strength (Unpaired *t*‐test, *t*_10_ = 2.819, *p* = 0.018; Figure 3d) in the PV‐cre group compared to the CONV group. However, the body weight (Figure 3b) and duration on the forelimb hang tests (Unpaired *t*‐test, *t*_10_ = 1.260, *p* = 0.2362; Figure 3e) or inverted grid (Unpaired *t*‐test, *t*_10_ = 2.001, *p* = 0.0732; Figure 3f) did not change significantly between the two groups. These results highlight the therapeutic potential of PV interneurons in restoring muscle atrophy compromised by cisplatin.

**FIGURE 4:**
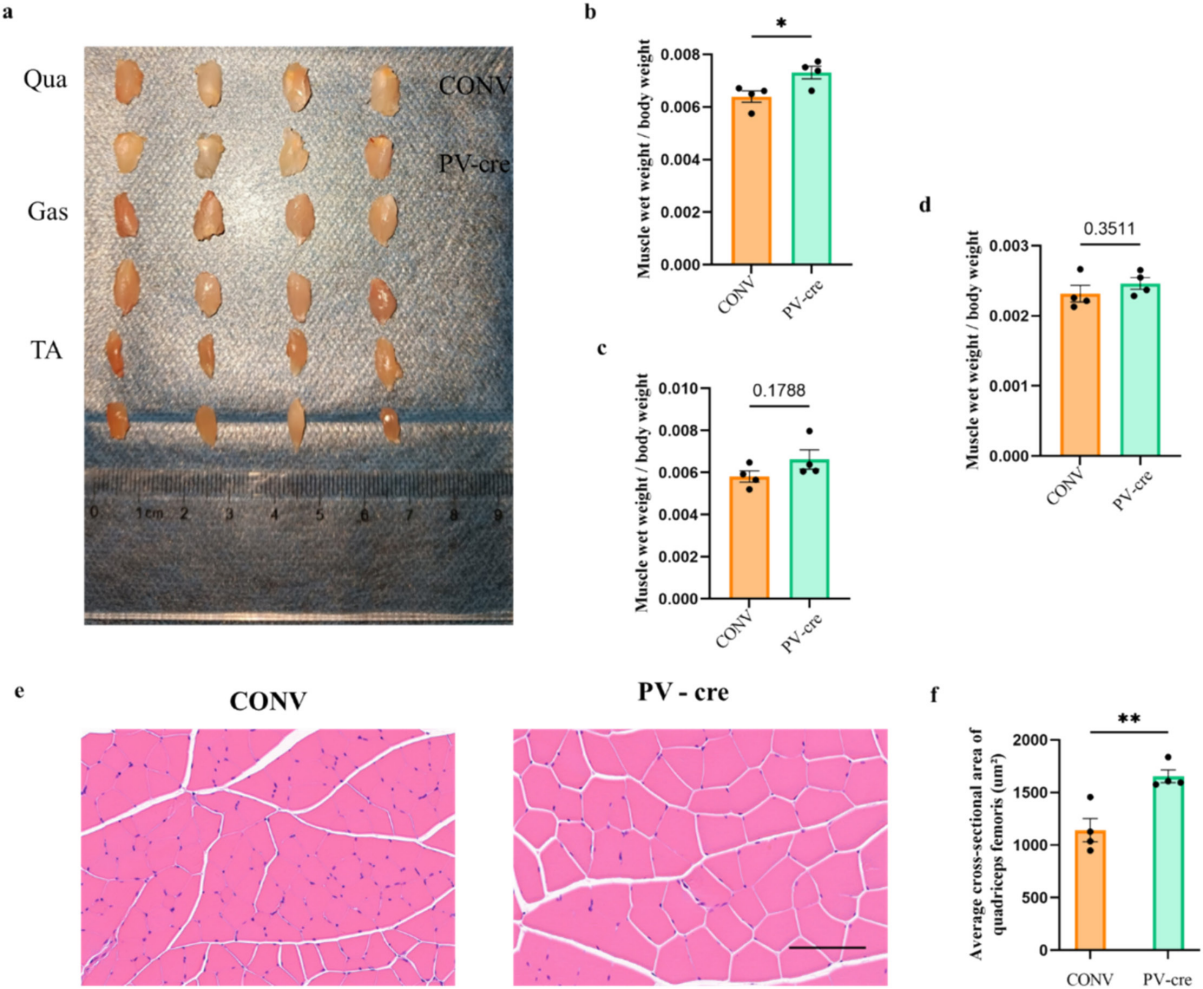
Changes in muscle mass and muscle fibre cross‐sectional area in cisplatin‐injected mice after chemogenetic activation. (a) Representative images of muscles from each group. (b–d) Muscle weight of the quadriceps (b), gastrocnemius (c) and tibialis anterior (d) were normalized to body weight. (e) Representative HE‐stained images of the quadriceps muscle (scale bar: 100 μm). (f) The cross‐sectional area of quadriceps muscle fibres was quantified using ImageJ software. Data are presented as Mean ± SEM (*n* = 4). CONV: control virus injection; PV‐cre: PVcre virus injection.

Histological analysis further supported these findings. Muscle wet‐weight ratios showed a trend of improvement, with a significant increase in quadriceps wet weight in the PV‐cre group (Unpaired *t*‐test, *t*_6_ = 2.818, *p* = 0.030; Figure 4a–d). Additionally, HE staining comparisons indicated an increase in fibre size, a reduction in fibre gap and fewer fibres per field in the PV‐cre group (Unpaired *t‐*test, *t*_6_ = 4.040, *p* = 0.0068; Figure 4e,f). The structural improvement suggests that PV interneuron activation ameliorates muscle atrophy not only by enhancing muscle function but also by promoting muscle fibre recovery.

The activation of PV interneuron addresses both motor deficits and muscle atrophy, which underscores its therapeutic potential in mitigating the side effects of cisplatin. These findings, combined with its protective effects on neuromuscular junction integrity, indicate that PV interneuron activation may serve as a promising intervention for chemotherapy‐induced muscle atrophy.

### 3.4 Short‐Term Exercise Mitigates Cisplatin‐Induced Muscle Atrophy

Given that PV interneuron activation demonstrated significant improvement in motor function, NMJ integrity and muscle structure, it was important to explore whether it achieves similar benefits to non‐invasive exercise intervention. PV interneurons play a critical role in regulating motor circuits, and their activation has been shown to alleviate spontaneous motor deficits caused by cisplatin. Since exercise is known to promote motor recovery and neuromuscular health by enhancing NMJ function and combating NMJ degradation [21], we hypothesized that it could serve as an alternative strategy to PV interneuron activation.

**FIGURE 5:**
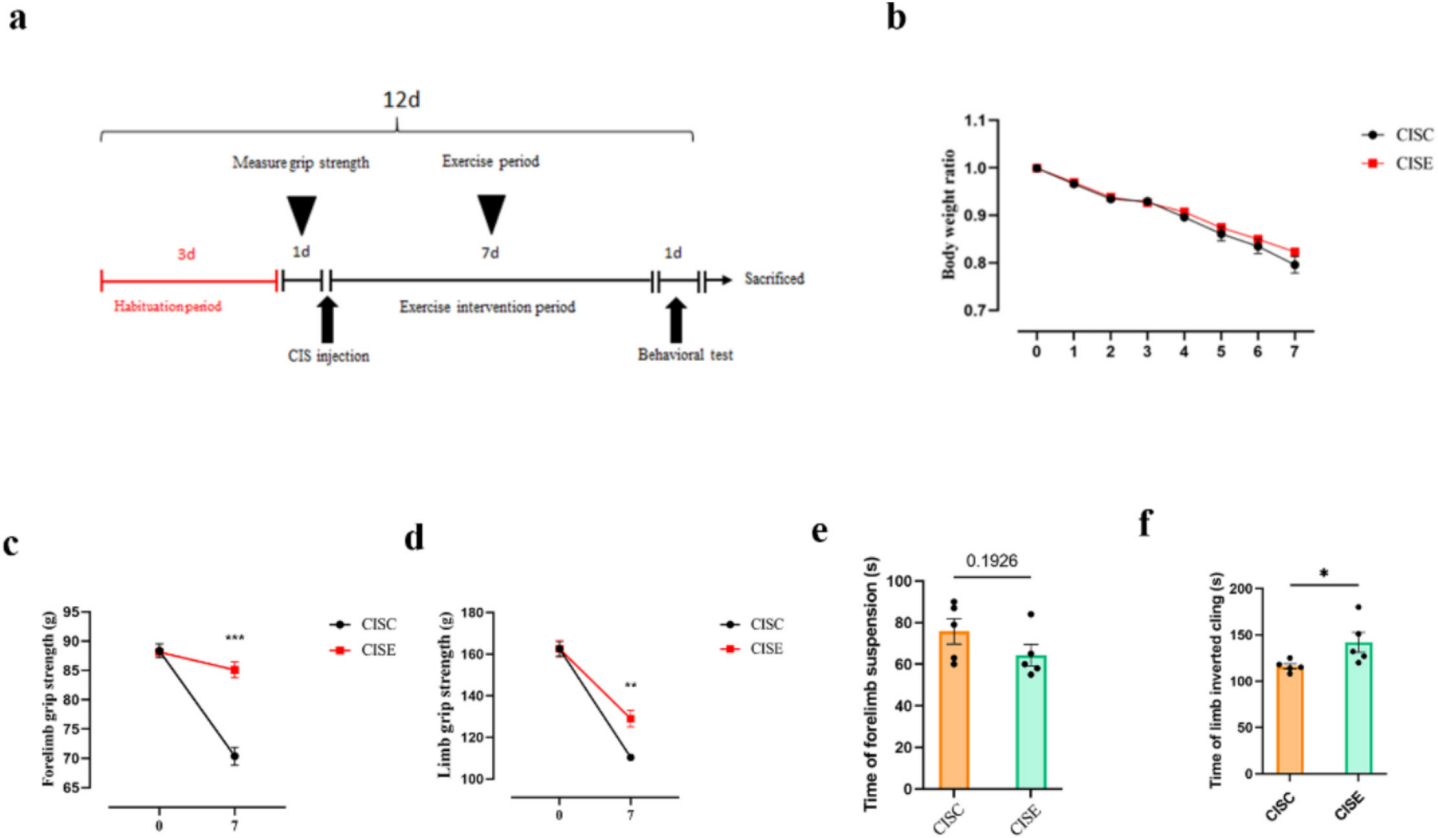
Effects of exercise on cisplatin‐induced muscle atrophy in vivo. (a) Schematic of the treadmill exercise intervention protocol. (b) Body weight ratio (measured weight/ initial weight) following exercise intervention in cisplatin‐treated mice. (c) Forelimb grip strength and (d) hindlimb grip strength assessed before and 7 days after exercise intervention. (e) Forelimb function and (f) hindlimb function were assessed by forelimb hang time. Data are presented as Mean ± SEM (*n* = 6). CISC: cisplatin‐treated sedentary control group; CISE: cisplatin‐treated exercise intervention group.

To evaluate this, cisplatin‐treated (CIS) mice underwent a short‐term treadmill exercise protocol following the induction of muscle atrophy. Behavioural and histological analyses were conducted to assess the impact of exercise on motor performance, muscle structure and NMJ integrity. Mice with cisplatin‐induced atrophy underwent exercise intervention (Figure 5a). While short‐term exercise did not significantly prevent cisplatin‐induced weight loss (*p* > 0.05; Figure 5b), it showed clear benefits for muscle performance. Exercise notably enhanced forelimb grip strength (Unpaired *t*‐test, *t*_8_ = 7.154, *p* < 0.001; Figure 5c), four‐limb strength (Unpaired *t*‐test, *t*_8_ = 4.526, *p* = 0.002; Figure 5d) and four‐limb hang time (Unpaired *t*‐test, *t*_8_ = 2.331, *p* = 0.048; Figure 5e,f), indicating enhanced muscle endurance and functional recovery.

**FIGURE 6:**
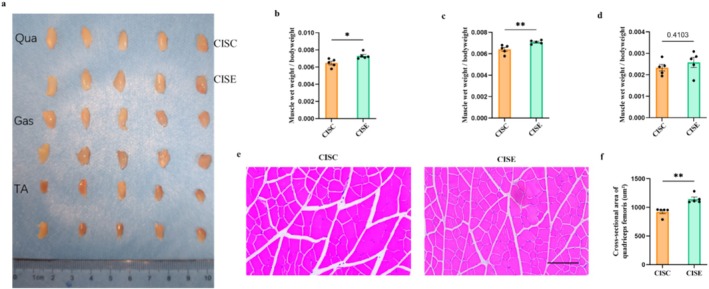
Effects of short‐term exercise on muscle mass and muscle fibre cross‐sectional area in cisplatin‐injected mice. (a) Representative images of muscles from each group. (b–d) Muscle weights of the quadriceps (b), gastrocnemius (c) and tibialis anterior (d) muscles normalized to body weight. (e) Representative HE‐stained images of the quadriceps muscle (scale bar: 100 μm). (f) The cross‐sectional area of quadriceps muscle fibres was quantified using ImageJ software. Data are presented as Mean ± SEM (*n* = 5, a). CISC: cisplatin‐treated sedentary control group; CISE: cisplatin‐treated exercise intervention group.

Histological analysis further confirmed the functional improvement. Muscle wet‐weight ratios of the quadriceps and gastrocnemius muscles were significantly higher in the exercise group compared to the untreated CIS group (Unpaired *t*‐test, *t*_8_ = 3.093, *p* = 0.015; *t*_8_ = 2.839, *p* = 0.022; Figure 6a–d), as well as in the average muscle fibre cross‐sectional area of quadriceps muscle (Unpaired *t*‐test, *t*_8_ = 4.569, *p* = 0.0018; Figure 6e,f), suggesting partial recovery of muscle structure.

These findings demonstrate that short‐term exercise effectively improves muscle strength and structure, even under the catabolic conditions induced by cisplatin. When combined with the results of activating PV interneurons, it becomes evident that PV interneuron activation offers an effective approach for mitigating cisplatin‐induced muscle atrophy, which is similar to exercise. Future studies should investigate the synergistic potential of combining exercise with neural interventions to maximize therapeutic benefits for chemotherapy‐induced muscle loss.

## 4 Discussion

This study demonstrates that cisplatin‐induced muscle atrophy and motor deficits in mice result from a combination of direct muscle toxicity, NMJ dysfunction and central motor regulation impairments. We further show that chemogenetic activation of striatal PV interneurons ameliorates these deficits by improving motor function, NMJ integrity and muscle structure. Additionally, short‐term exercise intervention has similar effects and partially restores muscle function and morphology. Thus, PV interneuron activation offers an effective approach for mitigating cisplatin‐induced muscle atrophy like exercise.

Cisplatin, a widely used chemotherapeutic agent, is known to induce severe systemic side effects, including muscle atrophy [18]. Chemotherapy‐induced cachexia and muscle atrophy are common side effects in cancer patients, adversely affecting their quality of life and the efficacy of chemotherapy and potentially reducing their lifespan. Abnormal weight loss often signals disease onset or progression and is a significant risk factor for increased mortality in cancer patients [22]. Muscle strength, mass and function are key indicators of muscle atrophy and NMJs play an important role in muscle health. Our findings highlight NMJ dysfunction as a critical contributor to muscle atrophy and motor impairments, aligning with previous studies [19].

In this study, we investigated the impact of activating striatal PV interneurons on cisplatin‐induced muscle atrophy in a murine model. Improvement in open‐field test parameters and the increased travel distance highlight the ability of PV interneurons to regulate motor circuits. Additionally, activation of PV interneurons enhanced NMJ integrity, as evidenced by a reduced ratio of partially innervated NMJs. These results extend the work of Gritton et al. [15], showing that sustained chemogenetic activation of PV interneurons can achieve effects similar to those observed with short‐term stimulation, positively influencing the motor state. However, it is essential to note that these findings are specific to cisplatin‐induced muscle atrophy in mice and may not be generalizable.

Additionally, relevant studies highlight the potential of exercise and neurogenesis in mitigating motor impairments. For example, Wei et al. demonstrated that treadmill exercise prevents motor function decline by blocking the complement pathway, counteracting the effects of clusterin release from adipocytes and alleviating TAR DNA‐binding protein 43 kDa‐induced motor impairments [23]. Further investigation revealed that running regulates PV interneurons in the hippocampus through PGC‐1α, reversing depression‐like behaviours [24]. Optogenetic activation of PV neurons can alter motor states, and exercise promotes adult neurogenesis, significantly increasing PV‐immunoreactive neuron protein levels after aerobic exercise programmes [25]. In line with these observations, Delgado‐Zabalza et al. indicated that PV neuron activity in the basal ganglia plays a crucial role in motor function regulation [26]. Thus, the activation of PV interneuron and exercise intervention may have alternative effects on the regulation of motor function.

The improvement in cisplatin‐induced behavioural phenotype observed in our study may be attributable to PV interneuron activation enhancing the striatal internal environment and promoting positive neurophysiological changes. Previous studies suggest that cisplatin contributes to striatal tissue deterioration, increasing markers of cellular stress and inflammation such as P53, nuclear factor kappa‐B pathway activation and tumour necrosis factor mRNA expression [27]. These molecular changes may impact PV interneuron activity in the striatum, as PV‐positive, fast‐spiking GABAergic interneurons are critical regulators within the striatal microcircuit. Within the striatum, PV‐positive interneurons, especially concentrated in the dorsolateral region, play pivotal roles in motor skill learning and conditioned responses to reward‐related cues [28]. Melzer et al. identified GABAergic cortical‐striatal projections, demonstrating differential regulation of striatal neurons by M1 and M2 somatostatin and PV cells, further emphasizing PV interneurons' regulatory impacts on striatal function [14]. In rodents, the dorsolateral striatum integrates excitatory inputs from the motor cortex and thalamus to regulate voluntary movement, producing incidental inhibitory outputs to other basal ganglia nuclei. PV neurons generate fast and reliable MSN inhibition in response to thalamic inputs and process excitatory inputs from the motor cortex both locally and plastically [29].

Furthermore, in basal ganglia‐related disorders, targeting parvalbumin‐expressing neurons has shown therapeutic potential. For example, activating PV neurons in the substantia nigra can restore motor function in Parkinson's disease models [26], and in a mouse model of cerebellar dystonia, activation of striatal parvalbumin interneurons, combined D1 receptor agonist and D2 receptor antagonist administration and selective ablation of striatal PV interneurons all alleviated involuntary movements [30]. In our study, long‐term chemogenetic stimulation of PV neurons in the striatum mitigated cisplatin‐induced muscle wasting and atrophy, extending previous findings on the short‐term effects of optogenetic stimulation on motor function. However, considering the specificity of chemotherapy drugs to cancer patients, further research should explore the effects of activating PV neurons in tumour‐bearing, aged and female models to determine whether this approach could alleviate chemotherapy‐induced muscle atrophy. The co‐administration of other drugs including corticosteroids, such as dexamethasone, which are routinely given with cisplatin to prevent nausea, also need further exploration.

Our findings showed improvement in activity distance with neuronal intervention with a concurrent reduction in the muscle‐related adverse effects induced by cisplatin. This aligns with recent studies indicating that exercise can effectively mitigate chemotherapy side effects, reduce cachexia and confer various benefits in cancer management and prevention [31]. Specifically, in an acute exercise intervention study with cisplatin‐treated mice, no significant differences in weight changes were observed between the control and exercise groups or between the cisplatin quiet control group and the cisplatin exercise intervention group [32]. However, exercise significantly enhanced muscle strength, alleviating strength losses associated with muscle atrophy and contributing additional positive effects [33]. Our results were consistent with most studies showing exercise's role in counteracting cisplatin's detrimental effects on muscle [32]. Although the underlying mechanism remains to be fully elucidated, it's possible that muscle sensitivity to exercise‐induced stimulation is heightened under cisplatin‐induced atrophic conditions. Furthermore, other contributing factors, such as nutrition, play a role in cisplatin‐induced skeletal muscle atrophy in murine models [34].

The distinct yet complementary effects of PV interneuron activation and exercise highlight the potential for an integrated therapeutic approach. While PV interneuron activation addresses central and neuromuscular coordination, exercise directly enhances muscle strength and structure. Combining these interventions could yield synergistic effects, optimizing therapeutic outcomes for cisplatin‐induced muscle atrophy and motor deficits. Future studies should explore whether different timing, dosage and sequence of these interventions could achieve combined efficacy. The molecular pathways underlying the protective effects of PV interneurons also remain to be fully elucidated. Investigating downstream signalling pathways and their interaction with NMJ and muscle‐specific factors would deepen our understanding.

In conclusion, our study highlights the multifaceted nature of cisplatin‐induced muscle atrophy, involving direct muscle toxicity, NMJ dysfunction and impaired motor regulation. Chemogenetic activation of PV interneurons effectively addresses these deficits, offering a novel neural‐targeted therapy. Exercise interventions enhance muscle performance and structure, providing a promising avenue for combined therapeutic strategies. Together, these findings lay the groundwork for future research into integrated interventions to mitigate the debilitating effects of chemotherapy‐induced muscle atrophy.

## Ethics Statement

All animal studies have been approved by the appropriate ethics committee and have therefore been performed in accordance with the ethical standards laid down in the 1964 Declaration of Helsinki and its later amendments.

## Conflicts of Interest

The authors declare no conflicts of interest.

## Supporting information


**Figure S1** Changes in muscle mass and muscle fibre cross‐sectional area after cisplatin administration. (a) Representative images of muscles from each group. (b) Representative HE‐stained images of the quadriceps muscle (scale bar: 100 μm). (c) The cross‐sectional area of quadriceps muscle fibres was quantified using Image J software. Data are presented as Mean ± SEM (*n* = 6). CON: control; CIS: cisplatin.
**Figure S2.** Open‐Field test results in cisplatin‐injected mice. (a) Movement trajectories in the open field for both CIS and CON groups. (b) Spontaneous motor activity was quantified as total travel distance (cm) within a fixed period. Data are presented as Mean ± SEM (*n* = 6). CON: control group; CIS: cisplatin‐treated group.
**Figure S3.** Impaired NMJs in cisplatin‐treated mice. (a) Representative images of NMJs in the TA muscle. The muscle fibres were stained whole mount with α‐BTX (red) to label AChR clusters and with NF/synapsin‐1 (green) to label nerve terminals. Indicated by triangles are representative impaired NMJs: fragmented receptors (bottom left), partially innervated NMJ (upper) and denervated NMJ (bottom right). Scale bar, 50 μm. (b) Quantification of NMJs in (a), including the ratio of fragmented receptors, partially innervated NMJs and denervated NMJs in each 20x field. CON group *n* = 199 NMJs from 6 mice; CIS group, *n* = 139 NMJs from 6 mice. Data are presented as Mean ± SEM (*n* = 6).
**Figure S4.** Colocalization of mCherry with PV protein in the striatum of mice. Scale bar, 50 μm. Indicated by triangles are representative PV neurons.

## References

[1] K. C. Huang , Y. F. Chiang , T. C. Huang , et al., “Capsaicin Alleviates Cisplatin‐Induced Muscle Loss and Atrophy In Vitro and In Vivo,” Journal of Cachexia, Sarcopenia and Muscle 14, no. 1 (2023): 182–197. 36401337 10.1002/jcsm.13120 PMC9891949

[2] S. Blauwhoff‐Buskermolen , K. S. Versteeg , M. A. de van der Schueren , et al., “Loss of Muscle Mass During Chemotherapy Is Predictive for Poor Survival of Patients With Metastatic Colorectal Cancer,” Journal of Clinical Oncology 34, no. 12 (2016): 1339–1344. 26903572 10.1200/JCO.2015.63.6043

[3] S. Peixoto da Silva , J. M. O. Santos , M. P. Costa E Silva , R. M. Gil da Costa , and R. Medeiros , “Cancer Cachexia and Its Pathophysiology: Links With Sarcopenia, Anorexia and Asthenia,” Journal of Cachexia, Sarcopenia and Muscle 11, no. 3 (2020): 619–635. 32142217 10.1002/jcsm.12528 PMC7296264

[4] Y. Shen , Q. Shi , K. Nong , et al., “Exercise for Sarcopenia in Older People: A Systematic Review and Network Meta‐Analysis,” Journal of Cachexia, Sarcopenia and Muscle 14, no. 3 (2023): 1199–1211. 37057640 10.1002/jcsm.13225 PMC10235889

[5] C. E. Orsso , M. Montes‐Ibarra , M. Findlay , et al., “Mapping Ongoing Nutrition Intervention Trials in Muscle, Sarcopenia, and Cachexia: A Scoping Review of Future Research,” Journal of Cachexia, Sarcopenia and Muscle 13, no. 3 (2022): 1442–1459. 35301816 10.1002/jcsm.12954 PMC9178172

[6] D. G. Candow , P. D. Chilibeck , S. C. Forbes , C. M. Fairman , B. Gualano , and H. Roschel , “Creatine Supplementation for Older Adults: Focus on Sarcopenia, Osteoporosis, Frailty and Cachexia,” Bone 162 (2022): 116467. 35688360 10.1016/j.bone.2022.116467

[7] Q. Xiang , Y. Hu , J. Zheng , W. Liu , and J. Tao , “Research Hotspots and Trends of Exercise for Sarcopenia: A Bibliometric Analysis,” Frontiers in Public Health 11 (2023): 1106458. 36969670 10.3389/fpubh.2023.1106458 PMC10031062

[8] Y. Yi , Y. Song , and Y. Lu , “Parvalbumin Interneuron Activation‐Dependent Adult Hippocampal Neurogenesis Is Required for Treadmill Running to Reverse Schizophrenia‐Like Phenotypes,” Frontiers in Cell and Development Biology 8 (2020): 24. 10.3389/fcell.2020.00024 PMC7010605 32117963

[9] A. C. Martel and A. Galvan , “Connectivity of the Corticostriatal and Thalamostriatal Systems in Normal and Parkinsonian States: An Update,” Neurobiology of Disease 174 (2022): 105878. 36183947 10.1016/j.nbd.2022.105878 PMC9976706

[10] J. He , M. Kleyman , J. Chen , et al., “Transcriptional and Anatomical Diversity of Medium Spiny Neurons in the Primate Striatum,” Current Biology 31, no. 24 (2021): 5473–5486.e6. 34727523 10.1016/j.cub.2021.10.015 PMC9359438

[11] R. M. Miralles , A. R. Boscia , S. Kittur , et al., “Parvalbumin Interneuron Impairment Causes Synaptic Transmission Deficits and Seizures in SCN8A Developmental and Epileptic Encephalopathy,” JCI Insight 9, no. 20 (2024): e181005. 39435659 10.1172/jci.insight.181005 PMC11529981

[12] S. Hijazi , A. B. Smit , and R. E. van Kesteren , “Fast‐Spiking Parvalbumin‐Positive Interneurons in Brain Physiology and Alzheimer's Disease,” Molecular Psychiatry 28, no. 12 (2023): 4954–4967. 37419975 10.1038/s41380-023-02168-y PMC11041664

[13] K. D. Milicevic , B. L. Barbeau , D. D. Lovic , A. A. Patel , V. O. Ivanova , and S. D. Antic , “Physiological Features of Parvalbumin‐Expressing GABAergic Interneurons Contributing to High‐Frequency Oscillations in the Cerebral Cortex,” Current Research in Neurobiology 6 (2023): 100121. 38616956 10.1016/j.crneur.2023.100121 PMC11015061

[14] S. Melzer , M. Gil , D. E. Koser , M. Michael , K. W. Huang , and H. Monyer , “Distinct Corticostriatal GABAergic Neurons Modulate Striatal Output Neurons and Motor Activity,” Cell Reports 19, no. 5 (2017): 1045–1055. 28467898 10.1016/j.celrep.2017.04.024 PMC5437725

[15] H. J. Gritton , W. M. Howe , M. F. Romano , et al., “Unique Contributions of Parvalbumin and Cholinergic Interneurons in Organizing Striatal Networks During Movement,” Nature Neuroscience 22, no. 4 (2019): 586–597. 30804530 10.1038/s41593-019-0341-3 PMC6744276

[16] M. Jendryka , M. Palchaudhuri , D. Ursu , et al., “Pharmacokinetic and Pharmacodynamic Actions of Clozapine‐N‐Oxide, Clozapine, and Compound 21 in DREADD‐Based Chemogenetics in Mice,” Scientific Reports 9, no. 1 (2019): 4522. 30872749 10.1038/s41598-019-41088-2 PMC6418145

[17] E. A. Elmorsy , S. Saber , R. S. Hamad , et al., “Advances in Understanding Cisplatin‐Induced Toxicity: Molecular Mechanisms and Protective Strategies,” European Journal of Pharmaceutical Sciences 203 (2024): 106939. 39423903 10.1016/j.ejps.2024.106939

[18] G. Sirago , M. A. Pellegrino , R. Bottinelli , M. V. Franchi , and M. V. Narici , “Loss of Neuromuscular Junction Integrity and Muscle Atrophy in Skeletal Muscle Disuse,” Ageing Research Reviews 83 (2023): 101810. 36471545 10.1016/j.arr.2022.101810

[19] Y. Miao , L. Xie , J. Song , et al., “Unraveling the Causes of Sarcopenia: Roles of Neuromuscular Junction Impairment and Mitochondrial Dysfunction,” Physiological Reports 12, no. 1 (2024): e15917. 38225199 10.14814/phy2.15917 PMC10789655

[20] Q. Wang , C. Cui , N. Zhang , et al., “Effects of Physical Exercise on Neuromuscular Junction Degeneration During Ageing: A Systematic Review,” Journal of Orthopaedic Translation 46 (2024): 91–102. 38817243 10.1016/j.jot.2024.03.007 PMC11137388

[21] J. Pratt , G. De Vito , M. Narici , and C. Boreham ,“Neuromuscular Junction Aging: A Role for Biomarkers and Exercise,” Journals of Gerontology. Series A, Biological Sciences and Medical Sciences 76, no.4 (2021): 576–585. 32832976 10.1093/gerona/glaa207

[22] P. D. Bonomi , J. Crawford , R. F. Dunne , et al., “Mortality Burden of Pre‐Treatment Weight Loss in Patients With Non‐Small‐Cell Lung Cancer: A Systematic Literature Review and Meta‐Analysis,” Journal of Cachexia, Sarcopenia and Muscle 15, no. 4 (2024): 1226–1239. 38650388 10.1002/jcsm.13477 PMC11294038

[23] J. A. Wei , L. Liu , X. Song , et al., “Physical Exercise Modulates the Microglial Complement Pathway in Mice to Relieve Cortical Circuitry Deficits Induced by Mutant Human TDP‐43,” Cell Reports 42, no. 3 (2023): 112240. 36924491 10.1016/j.celrep.2023.112240

[24] J. Wang , J. Tang , X. Liang , et al., “Hippocampal PGC‐1α‐Mediated Positive Effects on Parvalbumin Interneurons Are Required for the Antidepressant Effects of Running Exercise,” Translational Psychiatry 11, no. 1 (2021): 222. 33859158 10.1038/s41398-021-01339-1 PMC8050070

[25] B. Kim and H. I. Im , “Chronic Nicotine Impairs Sparse Motor Learning via Striatal Fast‐Spiking Parvalbumin Interneurons,” Addiction Biology 26, no. 3 (2021): e12956. 32767546 10.1111/adb.12956 PMC8243919

[26] L. Delgado‐Zabalza , N. P. Mallet , C. Glangetas , et al., “Targeting Parvalbumin‐Expressing Neurons in the Substantia Nigra Pars Reticulata Restores Motor Function in Parkinsonian Mice,” Cell Reports 42, no. 10 (2023): 113287. 37843977 10.1016/j.celrep.2023.113287

[27] Y. Chtourou , B. Aouey , M. Kebieche , and H. Fetoui , “Protective Role of Naringin Against Cisplatin Induced Oxidative Stress, Inflammatory Response and Apoptosis in Rat Striatum via Suppressing ROS‐Mediated NF‐κB and P53 Signaling Pathways,” Chemico‐Biological Interactions 239 (2015): 76–86. 26120027 10.1016/j.cbi.2015.06.036

[28] G. Chen , Y. Zhang , X. Li , et al., “Distinct Inhibitory Circuits Orchestrate Cortical Beta and Gamma Band Oscillations,” Neuron 96, no. 6 (2017): 1403–1418.e6. 29268099 10.1016/j.neuron.2017.11.033 PMC5864125

[29] Y. Nakano , F. Karube , Y. Hirai , et al., “Parvalbumin‐Producing Striatal Interneurons Receive Excitatory Inputs Onto Proximal Dendrites From the Motor Thalamus in Male Mice,” Journal of Neuroscience Research 96, no. 7 (2018): 1186–1207. 29314192 10.1002/jnr.24214

[30] T. Matsuda , R. Morigaki , H. Hayasawa , et al., “Striatal Parvalbumin Interneurons Are Activated in a Mouse Model of Cerebellar Dystonia,” Disease Models & Mechanisms 17, no. 5 (2024): dmm050338. 38616770 10.1242/dmm.050338 PMC11128288

[31] C. Zhu , H. Ma , A. He , Y. Li , C. He , and Y. Xia , “Exercise in Cancer Prevention and Anticancer Therapy: Efficacy, Molecular Mechanisms and Clinical Information,” Cancer Letters 544 (2022): 215814. 35803475 10.1016/j.canlet.2022.215814

[32] H. Sakai , M. Kimura , Y. Isa , et al., “Effect of Acute Treadmill Exercise on Cisplatin‐Induced Muscle Atrophy in the Mouse,” Pflügers Archiv 469, no. 11 (2017): 1495–1505. 28762162 10.1007/s00424-017-2045-4

[33] J. H. Bae , D. Y. Seo , S. H. Lee , et al., “Effects of Exercise on AKT/PGC1‐α/FOXO3a Pathway and Muscle Atrophy in Cisplatin‐Administered rat Skeletal Muscle,” Korean Journal of Physiology & Pharmacology 25, no. 6 (2021): 585–592. 34697269 10.4196/kjpp.2021.25.6.585 PMC8552830

[34] T. H. Hsu , T. J. Wu , Y. A. Tai , C. S. Huang , J. W. Liao , and S. L. Yeh , “The Combination of Quercetin and Leucine Synergistically Improves Grip Strength by Attenuating Muscle Atrophy by Multiple Mechanisms in Mice Exposed to Cisplatin,” PLoS ONE 18, no. 9 (2023): e0291462. 37699022 10.1371/journal.pone.0291462 PMC10497166

