# Supplementary material for: Activating Striatal Parvalbumin Interneurons to Alleviate Chemotherapy‐Induced Muscle Atrophy
Source: J Cachexia Sarcopenia Muscle. 2025 Apr 8;16(2):e13782. doi: 10.1002/jcsm.13782 (PMC11976163; doi:10.1002/jcsm.13782)
Supplement: Supplementary file 1 — Figure S1 Changes in muscle mass and muscle fibre cross‐sectional area after cisplatin administration. (a) Representative images of muscles from each group. (b) Representative HE‐stained images of the quadriceps muscle (scale bar: 100 μm). (c) The cross‐sectional area of quadriceps muscle fibres was quantified using Image J software. Data are presented as Mean ± SEM (n = 6). CON: control; CIS: cisplatin. Figure S2. Open‐Field test results in cisplatin‐injected mice. (a) Movement trajectories in the open field for both CIS and CON groups. (b) Spontaneous motor activity was quantified as total travel distance (cm) within a fixed period. Data are presented as Mean ± SEM (n = 6). CON: control group; CIS: cisplatin‐treated group. Figure S3. Impaired NMJs in cisplatin‐treated mice. (a) Representative images of NMJs in the TA muscle. The muscle fibres were stained whole mount with α‐BTX (red) to label AChR clusters and with NF/synapsin‐1 (green) to label nerve terminals. Indicated by triangles are representative impaired NMJs: fragmented receptors (bottom left), partially innervated NMJ (upper) and denervated NMJ (bottom right). Scale bar, 50 μm. (b) Quantification of NMJs in (a), including the ratio of fragmented receptors, partially innervated NMJs and denervated NMJs in each 20x field. CON group n = 199 NMJs from 6 mice; CIS group, n = 139 NMJs from 6 mice. Data are presented as Mean ± SEM (n = 6). Figure S4. Colocalization of mCherry with PV protein in the striatum of mice. Scale bar, 50 μm. Indicated by triangles are representative PV neurons. [file JCSM-16-e13782-s001.docx]

Appendix


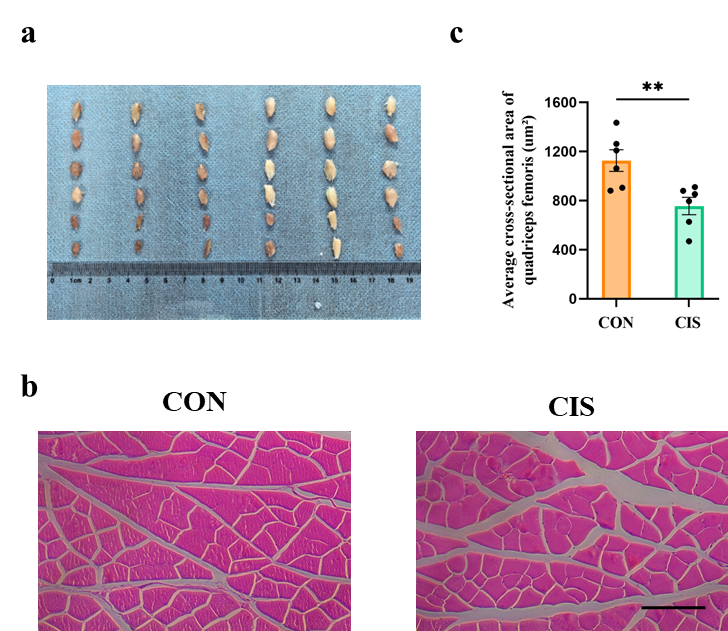


Figure S1 Changes in muscle mass and muscle fiber cross-sectional area after cisplatin administration. (a) Representative images of muscles from each group. (b) Representative HE-stained images of the quadriceps muscle (scale bar: 100 μm). (c) The cross-sectional area of quadriceps muscle fibers was quantified using Image J software. Data are presented as Mean ± SEM (n = 6). CON: control; CIS: cisplatin.


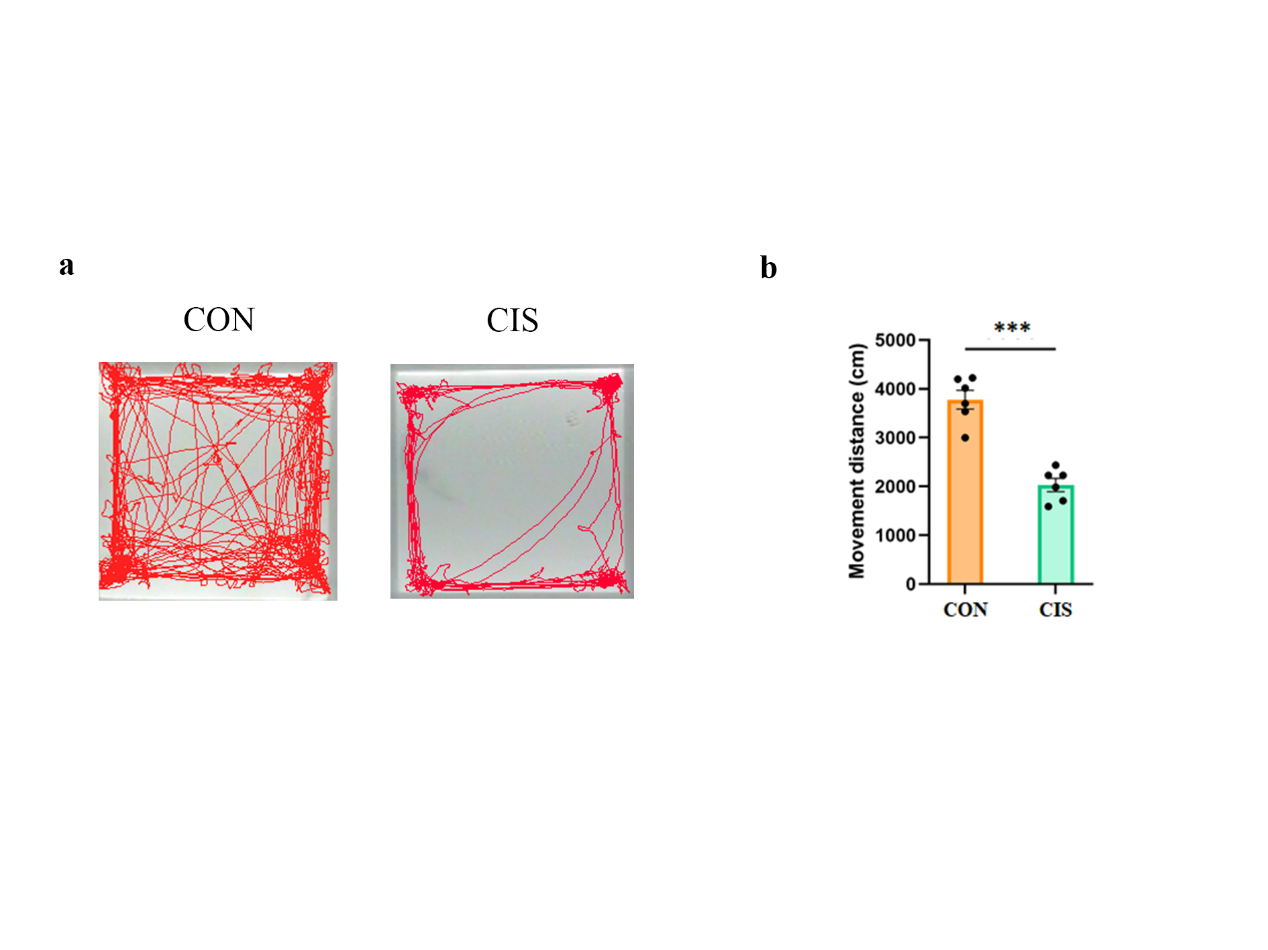


Figure S2 Open-Field test results in cisplatin-injected mice. (a) Movement trajectories in the open field for both CIS and CON groups. (b) Spontaneous motor activity was quantified as total travel distance (cm) within a fixed period. Data are presented as Mean ± SEM (n = 6). CON: control group; CIS: cisplatin-treated group.


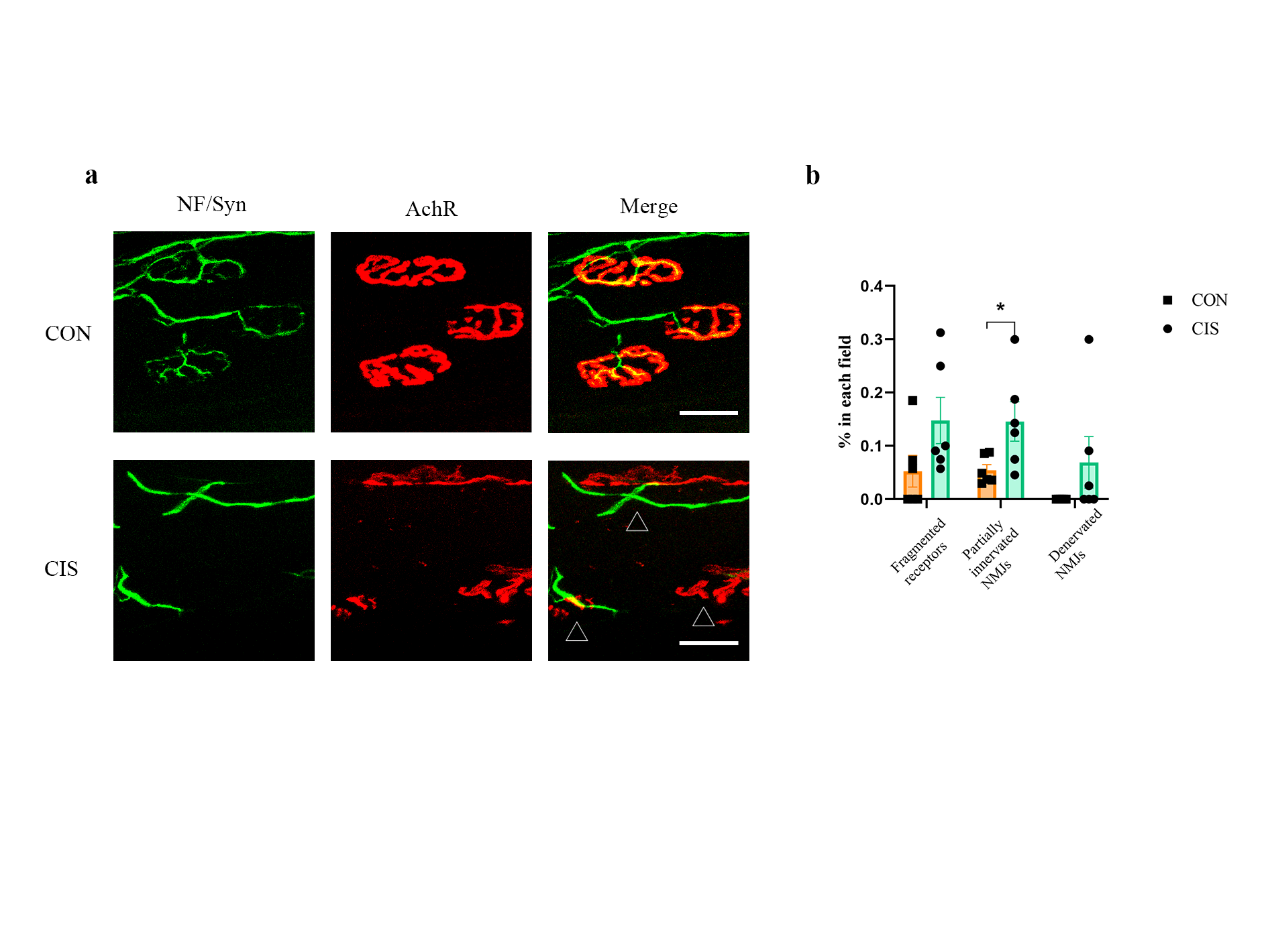


Figure S3 Impaired NMJs in cisplatin-treated mice. (a) Representative images of NMJs in the TA muscle. The muscle fibers were stained whole mount with α-BTX (red) to label AChR clusters and with NF/synapsin-1 (green) to label nerve terminals. Indicated by triangles are representative impaired NMJs: fragmented receptors (bottom left), partially innervated NMJ (upper), and denervated NMJ (bottom right). Scale bar, 50μm. (b) Quantification of NMJs in (a), including the ratio of fragmented receptors, partially innervated NMJs, and denervated NMJs in each 20x field. CON group n = 199 NMJs from 6 mice; CIS group, n =139 NMJs from 6 mice. Data are presented as Mean ± SEM (n = 6).


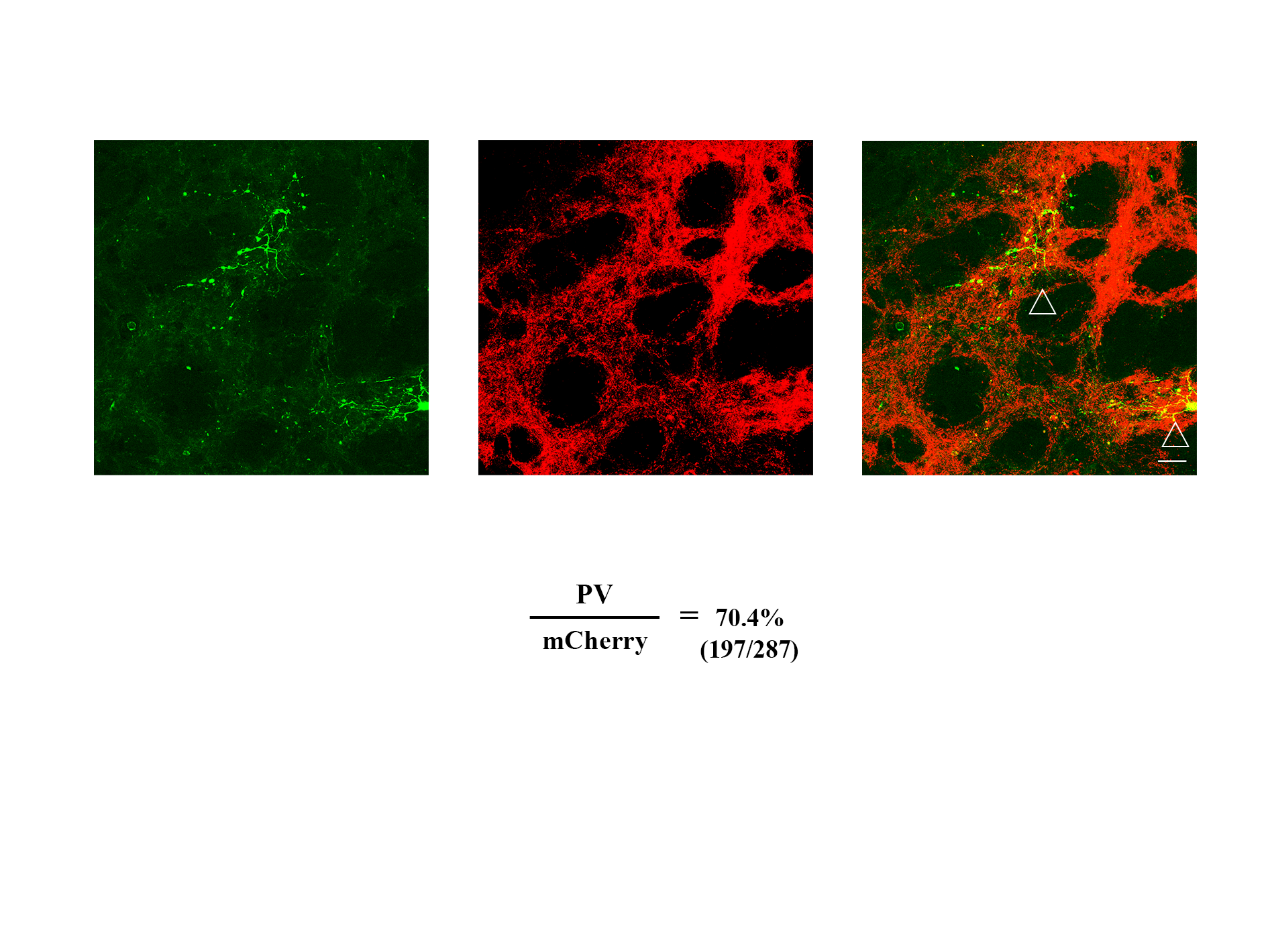


Figure S4 Colocalization of mCherry with PV protein in the striatum of mice. Scale bar, 50μm. Indicated by triangles are representative PV neurons.
